# Adolescent health experience after abortion or delivery (AHEAD) trial: formative protocol for intervention development to prevent rapid, repeat pregnancy

**DOI:** 10.1186/s12978-015-0098-4

**Published:** 2015-12-01

**Authors:** Michelle J. Hindin, Maria I. Rodriguez, Lianne Gonsalves, Lale Say

**Affiliations:** Department of Reproductive Health and Research including UNDP/UNFPA/UNICEF/WHO/World Bank Special Programme of Research, Development and Research Training in Human Reproduction, World Health Organization, Avenue Appia, 20, 1201 Geneva, Switzerland; Department of Obstetrics and Gynaecology, Oregon Health & Science University, 3181 SW Sam Jackson Park Road, Portland, OR 97239-3098 USA

**Keywords:** Adolescent, Pregnancy, Abortion, Intervention, Low-income countries, Middle-income countries

## Abstract

**Background:**

There is a high unmet need for modern contraception among adolescents, and adolescent girls who have already been pregnant are especially vulnerable to a rapid, repeat pregnancy (defined as a subsequent pregnancy within two years). The Adolescent Health Experience after Abortion or Delivery (AHEAD) trial will design, pilot, finalize, and ultimately evaluate an intervention targeted at reducing rapid repeat pregnancy. This protocol presents the methods for the first phase--formative research to identify key determinants of contraceptive use and rapid, repeat unintended pregnancy among adolescents.

**Methods/design:**

The determinants of adolescent pregnancy are known to vary by context; therefore, a dissimilar set of three countries will be selected to enable evaluation of the intervention in diverse cultural, political and economic environment, and to allow the intervention to be tested with a fuller range of ever-pregnant adolescents, including those who have chosen to terminate their pregnancy as well as those who are mothers. We will also consider marital status in settings where it is common for adolescents to marry. Focus group discussions (FGDs) will be conducted to examine barriers and facilitators to using contraception; preferred methods of overcoming these barriers; and perceptions of the services and information received. Key informant (KI) interviews will take place with various cadres of healthcare providers, health and education officials, and members of key youth and health organizations that work with adolescents. These interviews will focus on perceptions of pregnant adolescents; perceived information, skills, and motivations required for adolescent uptake of contraception; and experiences, challenges, and attitudes encountered during interactions.

**Discussion:**

The findings from this first formative phase will be used to develop an intervention for preventing rapid, repeat unintended pregnancy among adolescents. This intervention will be piloted in a second phase of the AHEAD trial.

## Background

The safety and efficacy of contraception for all women, including adolescents, has been well established [1]. However, high unmet need for modern contraception persists among adolescents in many low- and middle-income countries [2, 3]. A recent analysis of trends in unmet need for contraception among young women (ages 15–24) demonstrated high unmet need among with significant regional variations [2] Unmet need among sexually active, unmarried women ages 15–24 ranges from a low of 7.3 % in the Ukraine to a high of 69.5 % in Senegal [2].

Adolescents face a variety of interrelated barriers to contraceptive use. While many of these obstacles pertain to lack of access or knowledge about methods; social norms, provider misinformation and biases and expectations of adolescents can also be barriers to use [3–5]. Additionally, while an adolescent may want to space or delay pregnancy, in some settings, early marriage may mean that pregnancy is expected during adolescence. Interventions need to address the barriers adolescents face at the community, facility and individual levels. For example, at the community level, adolescents may want to “prove” their fertility or solidify a relationship with a pregnancy. Provider misperceptions regarding the safety or need for contraception by adolescents are barriers at the facility level [6] The adolescent herself may have fears or misunderstandings of the safety of contraception [7].

Adolescents who have had one unintended pregnancy are high risk for a rapid, repeat pregnancy (defined as a subsequent pregnancy within two years). Reported rates of rapid, repeat pregnancy among adolescents range from 20–50 % [8–11]. For some young women, a second pregnancy may be planned and desired. However, for many adolescents the second pregnancy is unintended. Contact with the health system for abortion, delivery and postpartum care provides an opening to recognize and respond to adolescent motivation to initiate contraception. It is paramount that these opportunities, whether part of routine or emergency care, are utilized to provide life-saving contraceptive services However, in many settings, the choice of methods is limited, and counselling and method provision is inconsistent Evidence suggests that postpartum and post-abortion use of longer acting methods (e.g. intrauterine device (IUD) and implants) is safe and effective [12–15], but often underutilized in low- and middle-income settings. These methods are also recommended for adolescents [16, 17].

Multiple interventions have targeted adolescent rapid repeat pregnancy. These interventions have included contraceptive provision, educational interventions (health education, HIV/STI prevention education, community services, counselling only, health education plus skills-building, faith based group or individual counselling), contraception promotion (contraception-education with or without contraception distribution) and complex interventions that contain multiple components [18–23]. However, definitive data on the efficacy of these interventions is lacking, particularly in developing countries.

There is an urgent need to provide guidance on effective strategies in developing countries that improve contraceptive use by adolescents and increase young women’s control over their reproductive health, in particular those at highest risk for unintended pregnancy (postpartum or post abortion adolescents) [24]. The Adolescent Health Experience after Abortion or Delivery (AHEAD) trial aims to address this evidence gap around effective interventions to avert rapid, repeat pregnancy among adolescents.

## Study objectives

Over three phases, in the AHEAD multi-country trial, we will design, pilot, finalize and ultimately evaluate an intervention targeted at reducing rapid repeat unintended pregnancy. In this protocol, we present the methods for the first phase: formative research to identify key determinants of contraceptive use and rapid, repeat unintended pregnancy among adolescents.

Phase 1 has three objectives:

- Objective 1: Identify key issues surrounding contraceptive use and prevention of unintended rapid repeat pregnancy (within two years).
- Objective 2: Determine the current health sector and community response to adolescent pregnancy including resources available to adolescents.
- Objective 3: Assess acceptability to adolescents and providers of potential intervention strategies to address unintended rapid, repeat pregnancy.

## Methods

### Study setting

Formative work will be carried out in each of the selected countries. The determinants of adolescent pregnancy are known to vary by context, and we want to ensure that the formative phase captures the range of barriers and enablers to effective adolescent contraceptive use in different settings. Within each country, sites will be selected based on volume and populations of adolescents presenting at facilities.

### Study design

In order to meet the objectives of this formative phase, we will conduct focus group discussions (FGDs) and key informant interviews (KIs), described below. The FGDs will be conducted by a trained focus group facilitator, and interviews by a trained interviewer. The FGDs will also be attended by a trained note taker, making notes of particular nonverbal cues or body language that could inform analysis. The FGDs will be conducted in the participants’ native languages. With consent, we will digitally record all interviews and then the transcripts will be translated and transcribed to English.

### Focus group discussions

Participants of the FGDs are the target group of the planned intervention: adolescents who have been pregnant. The FGDs will explore barriers and facilitators to using contraception; and what their preferred methods of overcoming these barriers would be. Further key areas to be explored in the qualitative part of the formative research are adolescents’ perceptions of the services and information they have received while utilizing these services.

### Key informant interviews

Key informant (KI) interviews will take place with various cadres of healthcare providers, health and education officials, members of key youth and health organizations that work with adolescents. The focus of these KIs will be on attitudes and beliefs about adolescent pregnancy and barriers and facilitators contraceptive use. Additionally, the KIs will also focus on obtaining their opinions, thoughts and perceived acceptability of potential interventions to prevent rapid repeat pregnancy.

### Study participants

We will recruit adolescents from health facilities. At the time when they will be seeking abortion or delivery care, they will be approached for potential participation.

Eligibility criteria for adolescents to participate in FGDs will be as follows:

- Adolescent girls between the ages of 15–19
- Presenting at participating facilities for abortions or delivery care

In settings where the intervention is likely to include post-abortion and post-partum adolescents, we will conduct separate focus groups to cover both populations.

Local PIs and the study team will identify providers at facilities, and the relevant holders that will be appropriate for the KIs.

### Participant recruitment

Recruitment will be based in facilities. Medical personnel in the wards in the facilities will be sensitized to the study and be asked to assist with recruitment. A research assistant will be available at the facility to discuss the study with any potentially eligible adolescents. The research assistant will explain the study and invite the adolescents to participate in a focus group at a later date. Once the adolescent expresses interest in the focus group, the research assistant will ask the adolescent for her contact information so she can be notified of the timing and the venue for the focus group. The contact information will not be retained after the focus group, and any adolescent who express concerned about being contacted will not be invited to the focus group.

Potential key informants will be contacted by the research assistants. Research assistants will describe the study and invite participation. If the person agrees, a mutually agreeable time and venue will be identified.

### Participant consent

Prior to beginning FGDs and KIs, research staff will obtain informed consent or assent from each participant. As part of the consent process, participants will be given more detailed information about the study background, goals, and objectives. Participants will be told that participation is free, voluntary, and confidential. All FGDs and KIs will be digitally recorded.

Adolescents and key informants aged 18 years and above will be asked to sign an informed consent but told that their names will not be linked to any of the information they provide, or written on any other form. Adolescents between the ages of 15–17 will be required to obtain parental or guardian consent from a parent or caregiver prior to participation. In cases where the adolescent has undergone an abortion and has not alerted a parent, we will use the local IRB recommendations on who should be allowed to give consent and whether the adolescent herself can give assent. While the names of participants will be recorded on the individual consent and assent forms, they will not be written on any FGD forms. During the conduct of the FGDs, participants will be given a number rather than using names. There will be no recorded linkage between the participants and identifying information-- anonymity of participants will be preserved.

### Sampling and allocation

We will use purposive sampling at selected facilities. The adolescents recruited as FGD participants will be separated into different discussion groups according to whether their pregnancy ended in a termination or birth.

### Sample size calculation

A minimum of two focus groups of 6 to 10 adolescents will be conducted per facility. Between four to eight facilities per country will be selected. The number of focus groups may change slightly across all sites in a selected country; if we continue to find new information after two per facility, we may conduct additional focus groups to ensure saturation. We will also conduct between 10 and 15 KIs per country.

### Study instruments

The focus group guide is vignette-based and the KI is semi-structured. The FGDs will focus on the following themes per participant category:

### Focus group discussion guide for adolescents

- Reactions to adolescent pregnancy (adolescents, partners, parents, health providers)
- Motivations for using or not using contraception
- Contraceptive methods available to adolescents and their awareness of them
- Experiences and relationships with health services
- Review and ranking of the participants’ perceptions of potential interventions to prevent unintended repeat pregnancy

### Key informant interviewer guides

- Reach of contraceptive services in the community, particularly for adolescents
- Type and quality of consultations with adolescents
- Community attitudes toward having adolescent pregnancy
- Recommendations to improve contraceptive services
- Exploration of interventions that could prevent unintended repeat pregnancy

### Data management

All FGDs and KIs will be conducted by trained research assistants in the preferred language of the participants. They will digitally recorded with the participants’ permission. All recordings will be stored locally on a secure server. Field notes will be transformed into data documents within a day of the KI or FGD. These notes, as well as transcriptions, will be anonymized, and no identifying information will be included in the notes.

Initially, KIs and FGDs will be transcribed verbatim in the original language. Following this, an independent translator will translate them into English. Digital versions of the transcripts will be kept for up to three years using a cloud-based storage that is password protected and only accessible by the local study team for each site (PI and research assistants) and WHO study team. The originals will be deleted off of the audio recorders following a data check to make sure the originals were uploaded to the cloud. No names will be included with the recordings.

All data collection and storage will be undertaken under the principle of strict confidentiality.

### Analysis

Data analysis will be conducted concurrently with data collection, to enable ending data collection at the point of data saturation. As the intention is to inform the development of an intervention, the analysis strategy must take this into account. Therefore, we will use framework analysis as analysis method [25]. Framework analysis contains the following steps:

- Familiarisation
- Identifying a thematic framework
- Indexing
- Charting
- Mapping and interpretation

Although there are distinct steps in the analysis, the analysis is iterative, going back and forth between the different steps, ensuring that the analysis is grounded in the data [25]. To start with, one analyst per team reads and re-reads the transcripts, to become familiar with the content. Thereafter, they will code the data, creating a coding matrix. This matrix will be checked by another, independent analyst, against the original transcripts. The analyst then goes back to the transcript, after changes have been made to the matrix, and codes transcripts according to the coding matrix. As coding continues, categories are developed, which can then evolve into themes.

The last two steps have also been described as developing descriptive and explanatory accounts of the data [26]. To develop a descriptive account, the researcher will write a description of the range of codes, categories and themes emerging from the data. This process requires constant referral to the original transcripts, to ensure accuracy. The explanatory accounts then involve making sense of the descriptive accounts, and interpreting findings in the context of literature from the systematic review, through comparing the concepts, codes and categories to existing knowledge.

We will hold a qualitative workshop to help the research assistants, PIs and team members be part of the process of developing in the intervention and work on peer-reviewed publications. WHO will co-host the workshop in collaboration with country teams.

### Ethics approval

The WHO HRP Review Panel on Research Projects (RP2), comprised of a committee of external reviewers, reviewed and approved the scientific and technical content of the study (protocol ID, A65984). We then obtained ethics review and approval from the WHO Research Ethics Review Committee (ERC).

### Study timeline

The time frame for the formative research and the development of the intervention is ten months. The preparation of tools for the formative phase and ethical review is expected to take approximately eight months. The estimated length for the entirety of Phase 1 is 18 months. Writing and dissemination will occur after the formative phase is complete.

## Discussion

### Expected study outcomes

Data collected will provide an in-depth knowledge of barriers to contraceptive use by adolescents; determinants of rapid, repeat, unintended pregnancy; and an assessment of potential interventions to increase contraceptive use among adolescents to prevent unintended rapid repeat pregnancies (less than two years). Additional anticipated outcomes are the identification of key issues within the health facility that restrict access to contraception for adolescents as well as an improved understanding of motivators and incentives for contraceptive behaviour change among adolescents and health care providers.

The findings from this first formative phase will be used to develop an intervention for preventing unintended rapid, repeat pregnancy among adolescents. This intervention will be piloted for feasibility and early proxy measures of efficacy in Phase 2 of the AHEAD trial.

### Contribution of the study to identifying and/or reducing inequities in sexual and reproductive health care

This protocol describes the first phase of a larger trial aiming to develop and test effectiveness of an intervention to reduce unintended repeat pregnancy in adolescent girls. The results of the trial are expected to benefit adolescent girls’ empowerment, meeting their needs and increasing their future prospects in life. It also will help increasing their access to health care and effective counselling for contraceptive choices.

### Plans for dissemination of research findings

At the conclusion of the study, a report will be prepared of the study findings. The report will be circulated to stakeholders participating in the study; an address for questions and follow-up will be included. Each primary investigator will be responsible for communicating with the sites regarding the findings. A formal manuscript will be prepared for publication in a peer reviewed journal and presentation at relevant academic conferences.

## Abbreviations

AHEAD: Adolescent Health after Abortion or Delivery
FGD: Focus Group Discussion
IUD: Intrauterine Device
KI: Key information Interview
PI: Principal Investigator

## References

[1] World Health Organization (2015). Medical eligibility criteria for contraceptive use.

[2] MacQuarrie K (2014). Unmet Need for Family Planning among Young Women: A Global Comparison of Levels, Trends and Components.

[3] Singh SD, J E (2012). Adding it up: the costs and benefits of investing in family planning and newborn and maternal health. Estimates for 2012.

[4] Wood K, Jewkes R (2006). Blood blockages and scolding nurses: barriers to adolescent contraceptive use in South Africa. Reproductive Health Matters.

[5] Castle S (2003). Factors influencing young Malians’ reluctance to use hormonal contraceptives. Studies in Family Planning.

[6] Chandra-Mouli V, McCarraher DR, Phillips SJ, Williamson NE, Hainsworth G (2014). Contraception for adolescents in low and middle income countries: needs, barriers, and access. Reproductive Health.

[7] Hindin MJ, McGough LJ, Adanu RM (2014). Misperceptions, misinformation and myths about modern contraceptive use in Ghana. J Fam Plann Reprod Health Care.

[8] Nelson PB (1990). Repeat pregnancy among adolescent mothers: a review of the literature. J Natl Black Nurses Assoc.

[9] Crittenden CP, Boris NW, Rice JC, Taylor CA, Olds DL (2009). The role of mental health factors, behavioral factors, and past experiences in the prediction of rapid repeat pregnancy in adolescence. J Adolesc Health.

[10] Milne D, Glasier A (2008). Preventing repeat pregnancy in adolescents. Curr Opin Obstet Gynecol.

[11] Raneri LG, Wiemann CM (2007). Social ecological predictors of repeat adolescent pregnancy. Perspect Sex Reprod Health.

[12] Grimes DA, Lopez LM, Schulz KF, Van Vliet HA, Stanwood NL. Immediate post-partum insertion of intrauterine devices. Cochrane Database Syst Rev. (5):CD003036. doi:10.1002/14651858.CD003036.pub2. 10.1002/14651858.CD003036 11406064

[13] Lopez LM, Hiller JE, Grimes DA, Chen M (2012). Education for contraceptive use by women after childbirth. Cochrane Database Syst Rev.

[14] Drey EA, Reeves MF, Ogawa DD, Sokoloff A, Darney PD, Steinauer JE (2009). Insertion of intrauterine contraceptives immediately following first- and second-trimester abortions. Contraception.

[15] Goodman S, Hendlish SK, Reeves MF, Foster-Rosales A (2008). Impact of immediate postabortal insertion of intrauterine contraception on repeat abortion. Contraception.

[16] Committee Opinion No. 539 (2012). Adolescents and long-acting reversible contraception: Implants and intrauterine devices. Obstetrics and Gynecology..

[17] Russo JA, Miller E, Gold MA. Myths and misconceptions about long-acting reversible contraception (LARC). Journal of Adolescent Health. 2013;52(4 Suppl):S14–21. doi:10.1016/j.jadohealth.2013.02.003. 10.1016/j.jadohealth.2013.02.003 23535052

[18] Drayton VL, Montgomery SB, Modeste NN, Frye-Anderson BA, McNeil P (2000). The impact of the Women’s Centre of Jamaica Foundation programme for adolescent mothers on repeat pregnancies. West Indian Med J..

[19] Daniel EE, Masilamani R, Rahman M (2008). The effect of community-based reproductive health communication interventions on contraceptive use among young married couples in Bihar, India. Int Fam Plan Perspect.

[20] Sebastian MP, Khan ME, Kumari K, Idnani R (2012). Increasing postpartum contraception in rural India: evaluation of a community-based behavior change communication intervention. Int Perspect Sex Reprod Health.

[21] Peipert JF, Madden T, Allsworth JE, Secura GM (2012). Preventing unintended pregnancies by providing no-cost contraception. Obstetrics and Gynecology.

[22] Olds DL, Henderson CR, Tatelbaum R, Chamberlin R (1988). Improving the life-course development of socially disadvantaged mothers: a randomized trial of nurse home visitation. American Journal of Public Health.

[23] Tu X, Lou C, Gao E, Shah IH (2008). Long-term effects of a community-based program on contraceptive use among sexually active unmarried youth in Shanghai, China. J Adolesc Health.

[24] Hindin MJ, Christiansen CS, Ferguson BJ (2013). Setting research priorities for adolescent sexual and reproductive health in low- and middle-income countries. Bulletin of the World Health Organization.

[25] Ritchie JS, L (2002). Qualitative data analysis for applied policy research. Analysing qualitative data.

[26] Smith J, Firth J (2011). Qualitative data analysis: the framework approach. Nurse Researcher.

